# Prevalence and determinants of osteoporosis in Congolese patients with axial rheumatism: a cross-sectional hospital-based study

**DOI:** 10.11604/pamj.2022.43.100.31519

**Published:** 2022-10-25

**Authors:** Aldo Mavinga, Jenny Mbuyi, Denis Matanda, Pierrot Lebughe, Jean-Christophe Mulumba, Viviane Nyembue, Adolphe Lukusa, Jean-Paul Divengi, Thierry Lusiensie, Jean-Marie Mbuyi-Muamba, Jean-Jacques Malemba

**Affiliations:** 1 Rheumatology Unit, Kinshasa University Hospital, University of Kinshasa, Kinshasa, Democratic Republic of Congo,; 2 Internal Medicine Unit, Sino-Congolese Friendship Hospital, Brazzaville, Congo,; 3 Internal Medicine Unit, Ngaliema Clinic, Kinshasa, Democratic Republic of Congo,; 4 Internal Medicine Unit, Provincial General Hospital of Kinshasa, Kinshasa, Democratic Republic of Congo

**Keywords:** Osteoporosis, axial pain, determinants, Kinshasa

## Abstract

**Introduction:**

osteoporosis seems to be uncommon in sub-Saharan Africa. The aim of this study was to determine prevalence and determinants of osteoporosis in Congolese outpatients attending rheumatology consultation for axial rheumatism in Kinshasa, Democratic Republic of the Congo.

**Methods:**

a cross-sectional hospital-based study from January to December 2018 among outpatients received for axial rheumatism in 8 hospitals of Kinshasa. The parameters of interest were age, sex, body mass index (BMI), alcoholism, smoking, physical activity, sunlight exposure, intake of dairy products, the notion of personal or parental fracture, a bone mineral density (BMD) and a phosphocalcic metabolism. The BMD was measured by using the dual energy X-ray absorptiométry. Serum level of calcium, Vitamin D, phosphore and parathormon were determined to assess the phosphocalcic metabolism. Osteoporosis was defined by a T-Score ≤ -2.5 SD. Standard statistical tests were used to analyze the results.

**Results:**

ninety patients (75 women and 15 men) were included. Their mean age was 63.5 ± 12.2 years. Low back pain 71.1% (n=63) was the main symptom. The rate of patients with osteoporosis, osteopenia, and normal bone density was 34.4% (n=31), 43.9% (n=44), and 16.7% (n=15) respectively. Pathological bone fractures were not noted. Ageing (aOR: 1.31, IC95%: 1.11-1.54; p=0.002), smoking (aOR: 14.65, IC95%: 1.38-156.1; p=0.045) and non-obese status (aOR: 32.3, IC95% 1.50-696; p=0.032) were identified as determinants of osteoporosis.

**Conclusion:**

in the present study, osteoporosis is common in Congolese patients with axial pain and is more frequent in women. Its determinants are ageing, smoking and non-obese status.

## Introduction

Osteoporosis is the commonly weakening disease of the skeleton. Because of its frequency as well as the high cost of its management, it constitutes a public health concern in developed countries [1,2]. The International Osteoporosis Foundation claims that at least one in three women and one in five men worldwide will suffer from this disease during their life [3]. The frequency of osteoporosis increases with age, principally over 65 years [4]. Taking into account the increase of life expectancy in developed countries, one can expect an increase in the frequency of osteoporosis and osteoporotic fractures during next decades [5]. The clinical and socio-economic burden of osteoporosis arises from the management of osteoporotic fractures [6]. The later are the basis of the morbidity and mortality due to osteoporosis [7,8].

In sub-Saharan Africa, few studies have been carried out on osteoporosis. This disease appears to be less common according to the literature because black people seem to be genetically protected by a higher bone mass when compared with Caucasian, Asian and Arab population [9]. In the Democratic Republic of Congo, a recent study found that references curves for the lumbar spine and total hip of Congolese patients are significantly different from the Caucasian, Asian or Arab normative data also explaining the low frequency of osteoporosis in our environment [10]. Based on this hypothesis born from the literature on the low frequency of this pathology in the Congolese environment as in sub-Saharan Africa, the aim of the present study was to determine the prevalence and to identify the determinants of osteoporosis in Congolese outpatients attending rheumatology unit for axial rheumatism.

## Methods

**Design, framework and population:** a cross-sectional hospital-based study was carried from January to December 2018 on outpatients with axial rheumatism attending the rheumatology units of some hospitals of Kinshasa. These hospitals selected were: Kinshasa Medical Center, University Hospital of Kinshasa, Diamant Clinic, General Provincial Hospital of Kinshasa, HJ Hospital, Monkole Hospital Center, Ngaliema Clinic and Polymedica Center. This selection was based on the organization within them of rheumatology consultations supervised by a rheumatologist. It reliably reflected rheumatological activity throughout Kinshasa, the capital and largest city of the Democratic Republic of the Congo.

**Selection and eligibility criteria:** patients were recruited comprehensively and consecutively to minimize selection biais and constitute the study population. The inclusion criteria were: any patient aged at least 40 years, consulting for low back pain and/or pelvic pain evolving for at least 3 months, having freely consented to participate in the study and having all our parameters of 'interest. None-inclusion criteria were: using, the last 3 months, drugs that may influence the bone mineral density (BMD), suffering from chronic pathology (hemoglobinopathy, neoplasia, chronic infection, hemopathy, endocrinopathy, nephropathy) that may lead to a secondary osteoporosis, immobilization period of more than 6 weeks during the previous 3 months as well as the presence of skin lesions or skin abrasions at the measurement site.

**Data collection:** all patients in the present study were examined by a rheumatologist. Lifestyle and physical examination data were recorded. The parameters of interest were age, sex, body mass index, alcoholism, smoking, physical activity, sunlight exposure, intake of dairy products, the notion of personal or parental fracture, a bone mineral density (BMD) and a phosphocalcic metabolism.

**Measurement of bone mineral density (BMD):** BMD was measured by dual-energy X-ray absorptiometry (DEXA) at HJ Hospital Kinshasa, a private center, the only one with this Hologic Discovery QDR 4500 brand bone densitometry device in the country. Examination carried out in a promotional context with the installation of this device. BMD was assessed at the lumbar column and hips for about 20 minutes. In case of contraindication for one of the 2 regions, the examination was performed at the distal region of the radius. BMD was expressed as absolute values (g/cm^2^) and as T- score. As per with the WHO recommendations, normal BMD, osteopenia, osteoporosis, and severe osteoporosis was a T-score >-1, -2.5 < T-score ≤ -1, T-score ≤ -2.5 and T-score ≤ -2.5 with pathological fractures respectively.

**Phosphocalcic metabolism assessment:** to assess phosphocalcic metabolism, two milliliters of blood were used for subsequent analysis of total serum calcium, phosphoremia, plasma vitamin D (25 OH Vitamin D), and parathyroid hormone using a SHIMADZU UV 1900 brand spectrophotometer at HJ Hospital on the same day of the bone densitometry making the possibility of missing data almost nil.

**Concepts' definitions:** the axial rheumatism was defined by the presence of low back pain or pelvic pain evolving for at least 3 months without any major trauma. Physical activities were considered as low if any physical activity was mentioned, moderate if physical activity duration was less than an hour per week and intense for vigorous practice that causes breath shortness, rapid heartbeat once or twice a week [11]. The frequency of milk intake by patients during the past 3 months was precised. It was considered as sufficient, or insufficient when taking milk at least twice a week and less than 2 times a week respectively [12]. It was considered as null when the patient did not take milk. Exposure to sunlight was assessed by the frequency of going outside the home over a period of a week and during the past 3 months. Going out of his home every day, at least three times a week, and less than 3 times a week was considered strong, moderate, and weak exposure to sunlight respectively [13]. Consuming at least 20 and 30g of alcohol a day defined alcoholism in women and in men respectively. Moderate drinkers were defined as people drinking less than 30g of ethanol a day for men and less than 20g for women, while heavier drinkers were defined as people drinking more than 30g of ethanol a day for men and more than 20g for women [14]. Smoking ≤ 5 and >5 packet-years (PUA) was considered as lower and heavier smoking respectively [15]. The body mass index (BMI) was measured and classified as needed using WHO recommendations; spinal deformities and fractures were looked for in the patients and their first-degree relatives.

**Statistical analyzes:** analyzes were performed using SPSS version 21.0 software. Data were presented as average ± standard deviations for continuous quantitative data, and as absolute or relative frequency for categorical variables. The Student's t-test was used to compare means and the Pearson's Chi-squared or Fisher's exact test as appropriate for proportions. Binary logistic regression was used to identify possible determinants of osteoporosis. The adjusted odd ratio values and 95% of confidence interval have been reported. The significant level was set at P-value < 0.05.

**Ethical considerations:** the data was collected anonymously and confidentially after the free and informed consent of each patient. The present study was approved by the national health ethical committee (CNES) of Democratic Republic of Congo with as approval number 361/CNES/BN/PMMF/2022.

## Results

**Figure 1:**
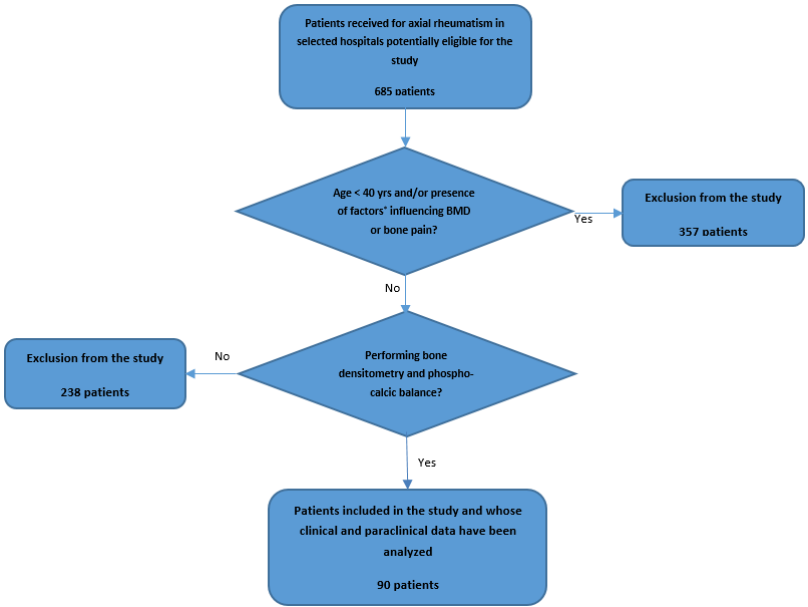
flow diagram showing numbers of patients at each stage of study

**General characteristics:** of the 685 patients with axial rheumatism recruited in the different selected hospitals (Figure 1), ninety patients (women: 83.3%, average age: 63.5 ± 12.2 years) were included in the present study and their general characteristics have been described in Table 1. The majority (52.2%) of them were recruited from the rheumatology unit of the University Hospital of Kinshasa. Overall prevalence of alcoholism, smoking, low activity, and overweight/obesity was 43.3%, 27.8%, 62.2%, and 74.6% respectively. The sunlight exposure was good for all the patients (Table 2).

**Table 1:** general characteristics of patients (n=90)

Variables			
Average age (years)		63.5 ± 12.2	
Age ranges (years) [n (%)]	40 - 49		18 (20)
	50 - 59		10 (11.1)
	60 - 69		22 (24.4)
	70 - 79		32 (35.6)
	80 +		8 (8.9)
BMI (Kg/m^2^)			27.0 ± 3.7
BMI ranges n (%)	Normal		23 (25.6)
	Overweight		46 (51.1)
	Obesity		21 (23.3)
Sex n (%)	Male		15 (16.7)
	Female		75 (83.3)
Menopause (n=75), n (%)			52 (69.3)
Menopause duration (n=52), n (%)	≥ 20 years		29 (55.8)
	< 20 years		23 (44.2)
Workers n (%)	yes		42 (46.7)
	No		48 (53.3)
Instruction level n (%)	None		12 (13.3)
	Primary		35 (38.9)
	Secondary		29 (32.2)
	University		14 (15.6)

Data were presented as averages with standard deviation, frequency and percentage. BMI: body mass index

**Table 2:** patients´ antecedents and life habits (n=90)

Variables		N	%
Alcohol consumption	No	51	56.7
	Moderate	20	22.2
	Heavy	19	21.1
Smoking	No	65	72.2
	Moderate	21	23.3
	Heavy	4	4.4
Physic activities	Low	56	62.2
	Moderate	21	23.3
	High	13	14.4
Milk consumption	No	13	14.4
	Low	39	43.3
	Sufficient	38	42.2
Bone fracture´s parent	No	80	88.9
	Yes	10	11.1

Data are presented as frequency and percentage.

**Clinical and densitometric's characteristics:** lumbar spine was the principal site (67.7%) of osteoporosis (Table 3). A normal t-score was noted in 15 patients (16.7%), an osteopenia in 44 patients (48.9%) and osteoporosis in 31 patients (34.4%). Osteoporosis was diagnosed in 29 women (38.7% of women and 93.5% of osteoporosis cases) and 2 men (13.3% of men and 6.5% of osteoporosis cases). So, the frequency of osteoporosis was 3 times higher in women than in men. One of ten patients reported a hip fracture in 1^st^ degree relative while none of them presented an osteoporotic fracture in the present study. Low back pain was the foremost symptom (71.1%). Osteoporosis was not an isolated condition in the present study; it was associated with osteoarthritis in 59 patients (Table 4).

**Table 3:** patients´ densitometric characteristics (n=90)

Parameters		Frequency	Percentage
Bone density	Normal	15	16.7
	Osteopenia	44	48.9
	Osteoporosis	31	34.4
Osteoporosis diagnosis´s site	Lumbar	21	67.7
	Femoral	2	6.5
	Lumbar and femoral	8	25.8
Osteoporosis and sex	Male (n=15)	2	13.3
	Female (n=75)	29	38.7

Data are presented as frequency and percentage

**Table 4:** patients’ clinical feature

Variables		N	%
Complaints	Low back pain	63	71.1
	Pelvic area pain	27	28.9
Pain type	Mechanical	57	63.3
	Inflammatory	21	23.3
	Neurogene	12	13.4
Associated disease (n=31)	Osteoarthritis	22	70.9
	Spondylarthritis	7	22.6
	Transitional abnomalitie	2	6.5

Data are presented as frequency and percentage.

**Phosphocalcic metabolism and osteoporosis associated factors:** the mean level of calcium, phosphore, vitamin D and parathormon were normal, while a deficiency of vitamin D was noted in one third of patients (Table 5). In the univariable analysis, ageing, smoking, non-obese status, menopause duration ≥ 20 years, parent's fractures were associated with osteoporosis. In the multivariable analysis, ageing (aOR: 1.31, IC95%: 1.11-1.54; p = 0.002), smoking (aOR: 14.65, IC95%: 1.38-156.1; p = 0.045) and the non-obese status (aOR: 32.3, IC95% 1.50-696; p = 0.032) were identified as independent determinants of osteoporosis in the present study (Table 6).

**Table 5:** parameters of phosphocalcic metabolism

		Average ± SD	n	%
Calcemia (mg/dl)		9.5 ± 0.4		
	Low		4	4.4
	Normal		84	93.3
	Hight		2	2.2
Phosphore (mg/dl)		3.4 ± 0.5		
	Low		6	6.7
	Normal		84	93.3
Parathormon (pg/ml)		68.9 ± 37.5		
	Normal		49	54.4
	Hight		41	44.6
Vitamin D (ng/ml)		33.6 ± 10.9		
	Low		32	35.6
	Normal		58	64.4

Data are presented as average, frequency and percentage

**Table 6:** determinants of osteoporosis

	Univariable analysis		Multivariable analysis	
	uOR (IC95%)	P	aOR (IC95%)	p
Age	1.20(1.10 - 1.31)	0.001	1.31 (1.11 - 1.54)	0.002
Female sex	3.7 (0.57 - 6.23)	0.077	-	-
Non-obese status	6.87(1.49 - 31.92)	0.006	32.3(1.50 - 696)	0.032
Menopause duration ≥20 ans	10.86(2.98 - 36.0)	0.001	-	-
Smoking	2.24(0.87 - 5.78)	0.041	14.65(1.38 - 156.1)	0.045
Alcohol consumption	2.50(1.03 - 6.10)	0.093	-	-
Physic activities	1.22(0.34 - 4.31)	0.763	-	-
Milk consumption	0.42(0.17 - 1.07)	0.066	-	-
Vitamin D deficiency	0.66(0.27 - 1.62)	0.359	-	-
A parent’s fracture	5.44(1.30 - 22.85)	0.01	-	-

Data are presented as Odds ratio and their confident intervals are reported. uOR : unadjusted odds ratio; aOR : adjusted odds ratio; CI : confidence interval

## Discussion

The present study aim was to determine prevalence of osteoporosis and its determinants in Congolese patients followed for axial pain. Results show that osteoporosis is common in these patients and is more frequent in women. Its determinants are ageing, smoking and non-obese status. The female predominance of osteoporosis is in agreement with the literature [16]. Since osteoporosis affects mostly women, several studies have been conducted only on women [17]. This female predominance might be explained by estrogen deprivation that occurs during menopause [18]. Two-thirds of the patients were at least 60 years-old. This is in line with the studies by Ka *et al*. [19] in Senegal, and Haouichat *et al*. [20] in Algeria who reported a average age over 60. Ageing is involved in the occurrence of osteoporosis through reactive hyperparathyroidism often due to a deficiency in calcium and vitamin D. This deficiency is due to an insufficient consumption of calcium and vitamin D, a lack of exposure to solar rays or even reduction of nephronic mass [21].

BMI is strongly associated to the occurrence of osteoporosis and osteoporotic fractures [22]. Indeed, the relative risk of occurrence of osteoporosis and osteoporotic fractures increases with a BMI ≤ 20 kg/m^2^ and tends to decrease with a BMI ≥ 30 kg/m^2^. The role of leptin in osteoblasts differentiation and estrogens aromatization that takes place in adipocytes may protect people with a high BMI from osteoporosis [23]. At least a third of the patients suffered from osteoporosis. This is the same as the studies by Aspray *et al*. [24] in Gambia (28.2%), Ka *et al*. [19] in Senegal (41.7%) and Ngandeu *et al*. [25] in Cameroon (13.6%). Contrastingly to our study, Tozin *et al*. [26] observed that postmenopausal Congolese women lost less bone mass per decade than Caucasian women. Kabeya-Kabenkama *et al*. [27] suggested that Congolese women attempted their peak of bone density later than Caucasians. These authors explained that this scarcity would be linked to a genetic protection which make that blacks would have a higher bone mass than other races. The African ancestral style of life, characterized by intense physical activity and significant exposure to sunlight, would also contribute to this protection [28]. The 27.7% smoking prevalence increased considerably the risk of osteoporosis. Tobacco decreases the half-life of circulating estrogens and impairs its release from adipose tissue. Ngandeu *et al*. [25] in Cameroon, also described an association between smoking and osteoporosis.

A third of our patients had vitamin D deficiency. This is in vein with the results by Ntyonga-Pono *et al*. [29] in Gabon (43.2% of patients) and Maataoui *et al*. [30] in Morocco (77.4% of patients). The vitamin D deficiency may be explained by the shelter effect of black skin which is rich in melanin, the clothing habits covering almost all from the body and an insufficient consumption of calcium and vitamin D. Concerning consumption of dairy products for example, 57.7% of patients reported having little or no milk consumption. No association has been found between the level of physical activity or alcohol consumption and osteoporosis.

The small size of the sample in the present study may explain the lack of association between other risk factors that are well known and closely linked to the occurrence of osteoporosis. The hospital nature of the study does not allow its results to be extrapolated to the general population. The credit for this study, however, is that it investigated osteoporosis using the benchmark tool and provided data showing that patients with axial rheumatism are candidates for osteoporosis.

## Conclusion

The present study suggests that osteoporosis is frequent in Congolese patients suffering from axial rheumatic disorders, with a strong female predominance. Severe osteoporosis was not observed among these patients. Ageing, smoking and non-obese status were retained as the determinants of osteoporosis.

### What is known about this topic

- Osteoporosis is a public health problem in developed countries because of its high frequency and the exorbitant cost of its management; in sub-Saharan Africa, Osteoporosis seems infrequent because Black Africans seem genetically protected by a high bone mass;
- The axial skeleton is a common site of osteoporotic fractures; the later are associated to a worsening mortality and morbidity in osteoporosis;
- The principals' determinants of osteoporosis are genetic factors, menopause, advanced age, sedentary life style, calcium and vitamin D deficiency.

### What this study adds

- Osteoporosis is frequent in Congolese patients with axial skeleton pain but fracture risk may be low;
- The use of DEXA highlighted the true extent of osteoporosis in the Congolese environment;
- Ageing, non-obese status and smoking are associated to osteoporosis in Congolese patients.
